# A Brief Review of Dental Associations

**Published:** 1859-01

**Authors:** 


### ARTICLE II.

*A Brief Review of Dental Associations.* By A SENIOR.

To argue that Dental Associations have contributed largely to the advancement which dentistry, both practically and scientifically, has made during the last twenty years would be to prove what every candid and intelligent member of the profession already admits; but that the organization and management of the several societies that have been formed during this period, have been free from errors, or have done all that they could do, no one will presume to affirm. Until the formation of the New York Society of Dental Surgeons a little more than twenty years ago, and which had only a very brief existence, the experiment of associated effort for the advancement of dentistry had never been tried. Indeed it would have been difficult at a very much earlier period to secure the co-operation of any considerable number of dentists, owing to the fewness of the members of the profession within convenient reach of any one place; and besides, the advantages of this peculiar specialty of physical alleviation had not become sufficiently established previous to this time to enable many to appreciate its importance. Hence, when the propriety of combined effort began to be agitated among dentists, it is not strange that the plans of organization fixed upon should be more or less imperfect, and as a consequence, the workings of the societies, rather desultory, than with definitely defined objects in view, or, if there were no diversity of opinion with regard to the latter, the means employed for their attainment were not always the best.

The New York Society, organized a little more than twenty years ago, owing to the want of a proper *esprit de corps*, among its members, was short-lived; yet the deliberations of the few meetings held, even by this association, were not wholly devoid of interest to the profession. Among the papers read at some two or three of its convocations, are several very creditable contributions to the literature of the profession, and if no very lasting or marked benefit resulted from it, it at least awakened a spirit of emulation among those who were immediately connected with it.

In August, 1840, a year or two after the dissolution of the New York Association, the American Society was formed. This was not intended to be local in its operations. The co-operation of the most intelligent members of the profession from every part of the Union was solicited, requiring, however, the recommendation of three as to moral character and professional ability, as a prerequisite to admission to membership. In three or four years, more than a hundred dentists were enrolled as members. Although voluntary and self-constituted, its workings were carried on under a constitution and by-laws adopted at the time of its formation. In August, 1841, at the first regular annual meeting, held subsequently to its organization in the city of New York, several interesting and valuable papers were read, and apart from the transactions of the regular meeting, about one day was devoted to the discussion of various methods of dental practice pursued at that time. It was at this meeting that the American Journal of Dental Science, which had now reached about the end of the first volume, was transferred to this association, and to the fostering care and support thus extended to it, its existence is mainly attributable. It remained the property of the society nine years.

The deliberations of the members of this association were characterized for some six or eight years by the utmost harmony and unanimity of feeling, exciting a spirit of inquiry among dentists all over the land, and thus an impetus was given to the advancement of dentistry, both as a science and art, which individual effort hitherto had failed to impart to it. It would be impossible to estimate the benefit that resulted, both directly and indirectly, from this great movement. The influence it exerted upon the profession is still felt; it gave rise to other organized movements—some, it is true, only of a temporary character, but others, which are still operative. The Virginia Society, the Mississippi Valley Association, the Pennsylvania Society, the Second New York Society, the Vermont Association, the New York State Society, as well as the North Carolina Association, the American Dental Convention, and lastly, the Northwestern and St. Louis Associations may all be regarded as offsprings of the American Society. To this list might also be added the Odontological Society of London, the College of Dentists of England, and perhaps it would not be going too far to mention the clause in the Medical Act recently passed by the British Parliament, granting to the Royal College of Surgeons, power to examine dentists and issue certificates as to their fitness to practice.* But whether the power, thus granted to the Royal College of Surgeons, if ever exercised, will be productive of any beneficial results, is, perhaps, after all, very questionable, as such examinations, if conducted solely by medical men, can only determine the medical qualifications of applicants, and a man may be thoroughly grounded in all the branches of general medicine and be profoundly ignorant of the practical details of the specialty for which he was examined.

To what extent the great movement originating in the formation of the American Society, may have contributed to the discoveries and improvements that have been made in the science and art of dentistry by individual members of the profession, even though having no connection with this or any other organized association of dentists, it is, of course, impossible to say, but if the truth of the matter were known, we think it very likely that many of them would be found to have their origin in the spirit of investigation and research to which it gave rise. This, and the other movements, started as a consequence of this, have no doubt scattered abroad germs of thought which have found lodgment in the minds of some, and perhaps without their being scarcely conscious of the fact, taken root, and years afterwards sprang up and bore fruit in the discovery of some valuable scientific principle, or the invention of some new and important method of practice. It is impossible to estimate the amount of good that grew out of the American Society and other more recently formed kindred associations. The influence even of those that have ceased to exist, is still operative, and is every day widening and extending itself, and it will continue to do so for years to come—giving rise to other and more wisely planned and better conducted

* Art. xlviii. It shall, notwithstanding anything herein contained, be lawful for Her Majesty, by Charter, to grant to the Royal College of Surgeons of *England,* power to institute and hold examinations for the purpose of testing the fitness of persons to practice as dentists, who may be desirous of being so examined, and to grant certificates of such fitness. *Act to regulate the qualifications of practitioners in Medicine and Surgery. 2d of August,* 1858.

movements; thus the spirit of improvement and investigation will be kept up until the dental specialty of medicine shall become as scientific in all its branches and be practiced by as accomplished men as ever adorned any calling or profession.

But apart from the direct influence exerted upon the profession by these societies, the demand for the services of the dentist has been greatly increased; not only by the additional consideration obtained for this peculiar medical specialty, but also from the greater benefit rendered suffering humanity as a consequence of the augmented skill of many of their members, arising from the free interchange of opinion, on the various methods of practice pursued by different dentists, and from the additional knowledge thus obtained. In consequence of this, respectable and well educated men have been induced to seek admission into the ranks of the profession by subjecting themselves to a more thorough and enlarged training than was formerly thought requisite. Thus the respectability and dignity of the calling has been elevated, its sphere of usefulness increased, and its resources enlarged. To every operation made by the dentist twenty years ago, nearly a hundred are now performed.

That all this change has been brought about through the agency of dental associations no one, it is presumed, is silly enough to suppose; but that it has contributed largely to it, every intelligent and candid member of the profession, who has carefully watched the progress of dentistry during this period, must admit. Other agencies, it is true, and to the existence and operation of some of which, the being of these are mainly, if not wholly, due, have at the same time, and were previously at work, and it may be that they contributed in a more eminent degree to it, than the society movement.

The publication of the American Journal of Dental Science was commenced more than a year before the formation of the American Society—the first number having been issued in June, 1839. The Baltimore College of Dental Surgery, the first institution of the kind ever established, was chartered in January or February, 1840, and went into operation the first of the following November. The Virginia Society was organized in 1843. The first and last number but one, of the British Quarterly, a dental journal, was published in March, 1844. The Mississippi Valley Association was formed the same year, and in 1844,–'5 the Ohio College was chartered. The publication of the London Forceps and Philadelphia Dental Intelligencer was commenced this year. The New York Dental Recorder made its appearance in 1846, and among the number of professional periodicals that have issued from the press subsequently to this period are, the News Letter of Philadelphia, the Obturator of New Orleans, the Review of St. Louis, the Reporter of Cincinnati, the New York Journal, L'Art Dentaire, the British Journal and the London Quarterly. In the meantime, the Syracuse, the Kentucky, the Philadelphia and Pennsylvania Colleges of Dental Surgery were established, although the school last mentioned is the only one of these institutions still in operation; several of the periodicals above named have also ceased to exist.

Although many, and perhaps most of these movements may have been unwise and premature as having had a tendency to cripple the energies, retard the progress and lessen the usefulness of others which, at the time were still in their infancy, by taking from them a portion, at least, of the support they then so much needed to enable them to develop into vigorous maturity and accumulate the resources they required for efficiency of operation; they, nevertheless, exerted some influence, and aided to some extent, at least, the progress of scientific and practical improvement. Thus, while they weakened and retarded the growth of other movements, it may be they produced good enough to counterbalance the injury that resulted from them.

The constitution and by-laws adopted by the American Society, at the time of its organization, for the regulation of its proceedings, would have insured the perpetuity of the association, had they always been rigidly enforced, but the provision relating to the admission of members, was never very scrupulously observed. Hence, many were brought within its portals utterly destitute of science and even of moderate excellence as practitioners; consequently they were incapable of aiding the promotion of any of the objects which the originators of the movement had in view in its formation—the advancement of dentistry and the elevation of its respectability. They were dead-weights to the enterprize, paralyzing the efforts of those who could, and were willing to work, many of them being influenced by no other motive than the consideration which they hoped to derive from association and fellowship with those who from more creditable attainments, enjoyed the respect of men of science and the confidence of such as needed the professional aid of the dentist. The admission of one such rendered the introduction of others of like claims easier, and thus in a few years, the names of many were enrolled among the members; so that the transactions of the society, after the first eight or ten meetings, instead of increasing in interest and enriching more and more the archives of the profession with additional scientific truths, discoveries and improvements in practice; each succeeding convocation, from this time, became less and less instructive, until finally, the association, from mere inanition, ceased to exist. To the introduction even of tyroes and men of very moderate professional ability into a society of this kind, whose sole ambition is the acquisition of knowledge with a view to greater usefulness, might not, perhaps, be very objectionable, but no scientific body could profit much from the membership of such individuals, and the admission of ignorant, self-important, meddling men to fellowship and participation in the proceedings of such an association is dangerous, if not fatal, to the harmony of its deliberations.

But the errors committed by the American Society may have been unavoidable, for at the time of its formation, and even ten years later, the number of scientific men in the profession, was smaller than at present, and not many had at that time any experience in the workings of deliberative bodies. It is easier to see the errors now than it was before they were committed, and those who did foresee them, were unable to convince others that they would prove such, until the rubicon was passed. It was then too late to retrieve them ; discord was introduced, and in a few years the society was dissolved. Whether it fulfilled its mission or not, it certainly accomplished much good, and there are many now who look back with feelings of delight to the meetings of the first ten years of its existence. These annual reunions of its members will ever be remembered by the writer with emotions of the liveliest pleasure. Friendships were formed at these meetings that will be cherished by many to the latest period of life, and if bitter enmities were contracted in consequence of words spoken during the excitement of debate, or of differences of opinion, it is hoped they are no longer remembered, or if remembered, have long since given place to better and kinder feelings.

But before the dissolution of the American Society another national association was organized under the name of the "American Convention of Dental Surgeons," but without constitution or laws, and which freely admitted all claiming to be practicing dentists, to participation in its proceedings. The propriety of this movement is regarded by many as questionable, and so far as the originators of the scheme had in view the advancement of dentistry as a science, very little good, it is feared, will be accomplished. Still, by bringing dentists together from different parts of the country once a year, it gives them an opportunity of interchanging professional views, and thus many, no doubt, are benefited. The discussion of subjects belonging to the daily occupation of men of the same calling, cannot be otherwise than interesting, especially to those who take part in it, and it is instructive in proportion as the speakers are well informed individuals. There is, however, one great objection to admitting all to membership without regard to professional or moral fitness, who may happen to apply. By taking into fellowship an ignoramus of vile character, and this is virtually done if admitted, the mere fact gives him, in the estimation of the world, a consideration to which he is by no means entitled. It is neither just to the profession, nor to the community to do so; the fact of his being a member of a society of this kind, is received by many as *prima facie* evidence of professional ability, enabling him to impose upon the unsuspecting, and subjecting his calling wherever he may happen to be, to unjust obloquy and opprobrium—an evil which the convention, as at present constituted, cannot prevent, and which deters many respectable dentists from attending its meetings, because they are unwilling to subject themselves to the liability of contact with individuals, *claiming brotherhood*, of doubtful reputation, with whom they would not elsewhere associate. If none such have as yet attended the meetings, it is by no means certain they never will. Thus far, it is true, the meetings of the convention have been attended by many of the most respectable members of the profession, who have participated freely in its deliberations. But others, whose coöperation it would be desirable to have, for reasons above stated, have refused to identify themselves with the movement. Whether they have acted wisely in the course they have pursued, it is perhaps, somewhat questionable. Had they entered heartily and cordially into it, the objectionable features complained of might never have existed. A wider field of inquiry, too, might have been opened to the investigations of the convention, and a better and more thorough method of conducting them been devised ; and hence they have lost rather than gained by staying away. Still, they believe they have acted rightly, and the sooner the convention adopts a constitution and laws that shall prevent the admission of unworthy individuals, the better.

Of the local society movement, the Mississippi Valley Association, having been longer in operation, has probably exerted a wider influence upon the profession than any other. Its annual meetings, have, for the most part, been well attended, and the discussions on these occasions have been interesting and more or less instructive. In the published transactions, which it has from time to time sent forth, are several very creditable contributions to the literature of dentistry. But the labors even of this association have not been sufficiently directed to a thorough, systematic investigation of the various subjects that have engaged the attention of its members. They have been too much confined to giving expression to opinions not always carefully formed; still the profession throughout the Mississippi valley, as well as in other parts of the Union, has been benefited by them.

The Virginia Society, formed before the Mississippi Valley Association, held a number of annual meetings, and at three or four of which interesting papers were read, and judging from the ability of several of its members, the discussions on these occasions were no doubt both interesting and instructive. The society is still in existence, but has not, so far as the writer is informed, held a regular annual meeting for several years. It has a charter from the legislature of the state, investing it with authority to grant degrees of doctor of dental surgery. But the propriety of exercising such powers by any other body than the proper authorities of a regularly constituted school of instruction, where the several branches of knowledge necessary to constitute a scientific practitioner of dentistry, are taught, is very questionable, and so thoroughly convinced of this were a number of the members of the association, that it was only done in a very few instances.

It is hoped that the meetings of this association will soon be resumed, and that the members will continue the contributions of their experience and research to the rapidly increasing general stock of professional knowledge. Having commenced a good work, it would be a pity for them to stop, as their labors with accumulating experience and scientific information, becomes every year more and more valuable to the world.

The Second New York Society, from the time of its organization, met very frequently, we believe, for several years. The meetings too, are said to have been well attended, and the discussions at many of them highly interesting, but for some cause or other, we believe they are now less frequent. This ought not to be. In a city like New York, with nearly three hundred dentists, and among the number many very able men, more ought to be done for the advancement of dentistry than in any other one place in America. Of the general character of the proceedings of this society, the writer can say but little, having only seen those of a few of its meetings, and in these there was the result of no elaborate systematic investigation of any one subject.

The interest of the meetings of the Pennsylvania Association, has, for the most part, we believe, been very well sustained from the beginning; and, although it does not appear from the published transactions, that it has contributed much by way of memoirs or scientific reports of committees, to the literature of the profession, yet the movement has been attended with beneficial results. It has brought many of the dentists of Philadelphia, and some from other parts of the State, frequently together, and established a more friendly intercourse between them than formerly existed. Thus, in a social and practical point of view, it no doubt, has done much good.

The doings of the Vermont Association, organized in 1854, and the New York State Society, at an earlier period, also constitute a part of the general movement of the profession, which had its origin in the formation of the American Society, but as the proceedings of the meetings of these bodies have not appeared regularly in any of the dental journals of the day, little is known, except by the members, of what they have done. There has not been a meeting of the last named association, so far as the writer is informed, for many years.

The meetings of the Northwestern and St. Louis Associations, formed some two or three years ago, have thus far been well attended, and some of the discussions on these occasions were highly interesting, indicating on the part of several of the speakers, a thorough acquaintance both with the practical and theoretical details of the profession. These associations have already awakened a spirit of emulation and inquiry among the dentists of the far west, that cannot be otherwise than productive of valuable results.

The two rival associations recently organized in England—the College of Dentists and the Odontological Society, have already given many valuable contributions to the literature of dental science, and the writer of this article takes pleasure in acknowledging his indebtedness to his transatlantic brethren. May the only rivalry between them be to do the most for the promotion of the objects for which they were formed; the future will then realise the fair promise of their beginnings.

In the foregoing brief review, the writer may not always have been correct with regard to dates and some other facts, having relied mostly upon his recollection of these as they occurred, and he has confined his remarks chiefly to the American Society and the American Convention, because these associations have exerted a wider influence upon the profession than those of a more local character. If neither thus far has adopted the best means for the accomplishment of the ends proposed, the fact is not alluded to in any unkind spirit, but in the hope that measures may be taken by the associations now in existence, to secure a thorough investigation of all subjects connected either with the theory or practice of dentistry, which may hereafter come before them for consideration. This can only be done by referring them to committees of competent men who are willing to bestow upon them the time and labor necessary to enable them to arrive at enlightened conclusions, with instructions to report fully the results of their investigations. One such report would throw more light on any given subject than a month's discussion by fifty individuals who had not previously made themselves equally well acquainted with it. If, in the investigation of the subject, chemical or other experiments, involving an outlay of money are required, this at least, should be furnished, and should compensation for the labors of the committees be necessary, it also should be made.

Each association should create a special fund to be given as awards for valuable improvements in practical dentistry, and for contributions to the science and literature of the profession. This might be done by the payment of three or five dollars a year by each member. These awards, of course, should be judiciously made, and never bestowed where they were not merited. They might be given either in money or gold medals. To gain a testimonial of this kind, many of the most talented dentists in our land would labor diligently for months and perhaps years ; but the competition should not be restricted to members of the profession in the United States alone ; it should be open alike to all everywhere. No matter where the contribution comes from, if it is really valuable, the author should receive an award, as far as circumstances will permit, proportionate to its worth.

If our associations would encourage genius and talent in this way, dentistry, both as a science and art, would advance more rapidly. Other professions do it, and why should not the dental? It has not yet reached the *ne plus ultra* of perfection, and as it is as intimately connected with the alleviation of human suffering as any of the other specialties of medicine, no efforts should be spared to increase its efficiency.

